# Clinical application of pedicled thoracoacromial artery perforator flaps for tracheal reconstruction

**DOI:** 10.1186/s12893-020-00972-9

**Published:** 2020-11-25

**Authors:** Di Deng, Feng Xu, Jifeng Liu, Bo Li, Linke Li, Jun Liu, Fei Chen

**Affiliations:** Department of Otorhinolaryngology Head & Neck Surgery, West China Hospital, Sichuan University, No. 37 Guo Xue Xiang, Chengdu, 610041 Sichuan P.R. China

**Keywords:** Pedicled thoracoacromial artery perforator flap, Tracheal reconstruction, Double-island flap, 2-stage, Thyroid carcinoma

## Abstract

**Background:**

Large or complex trachea defects often require some tissue to reconstruct, various flaps have been reported for reconstructing this defect. However, pedicled thoracoacromial artery perforator flap have not been reported in tracheal reconstruction. Therefore, this study is to assess the efficacy and clinical application of pedicled thoracoacromial artery perforator flaps for tracheal reconstruction.

**Methods:**

Eight patients who underwent tracheal reconstructions with pedicled TAAP flaps between December 2017 and October 2019 were retrospectively reviewed.

**Results:**

All of the pedicled TAAP flaps in our study survived. The flap size ranged from 2 cm × 5 cm to 4 cm × 10 cm, and the size of each island of one double-island flap was 2 cm × 2.5 cm. The mean thickness was 0.6 cm, and the pedicle length varied between 6 and 9 cm (mean 7.9 cm). The mean time of flap harvest was 17 min. The mean age of the patients was 62.4 years and five elderly patients had comorbidities, such as diabetes, hypertension and asthma. One patient received a double-island flap for tracheal and esophageal reconstruction, and the other patient received simple tracheal reconstruction. One patient died due to cancer metastasis. Six patients obtained functional recovery of breathing, except one patients who did not experience closure of the tracheostomy opening due to uncompleted I131 treatment.

**Conclusion:**

Pedicled TAAP flaps provide a short harvesting time, thin thickness and stable blood supply, and they do not require microsurgical skills. This flap is a good choice for the reconstruction of tracheal defects, especially in the aged or patients with comorbidities who are not able to tolerate prolonged surgery.

## Background

Tracheal defects can be caused by primary or invasive tracheal tumors, tracheal trauma, tracheal stenosis, or tracheal-esophageal fistula. Reconstruction of tracheal defects has received increasing attention from clinicians. There are various methods for repairing tracheal defects, including the use of end-to-end anastomoses, tissue-engineering materials, allografts, and autologous tissues. End-to-end anastomosis is a simple method for repairing tracheal defects, but it is mainly limited by the length of the defect [1]. Engineered airway transplantation seems to be a comprehensive method; defects can be reconstructed by “new airway” vascularization and canalization by seed cells, tracheal stents and growth factors, but this method is rarely used in the clinic because of issues with compatibility, complications and technical difficulties [2, 3]. Allograft or trachea transplantation cannot be popularized due to the long and complicated preparation period and the high technical requirements [4, 5].

In the clinic, a common way to reconstruct tracheal defects is via self-tissue. Due to their relatively simple preparation, high survival rate, and limited complications, self-tissue methods such as the use of pericardium flaps, autologous tissues, and flaps with autologous cartilage can be used to reconstruct defects [6, 7]. Flaps represent one of the common reconstruction methods, but there are many types of flaps. According to different donor areas, the texture, thickness, hair growth and other conditions of skin flaps are different as are the complications associated with the donor area. Therefore, each flap has its own advantages and disadvantages in tracheal reconstruction.

The thoracoacromial artery is the branch of the axillary artery at the upper margin of the pectoralis minor [8], and the pedicled thoracoacromial artery perforator (TAAP) flap is a popular flap used for the reconstruction of head and neck defects. Owing to the adequate color matching, texture, and pliability in combination with the limited anterior chest wall donor site morbidity, pedicled TAAP flaps can be successfully used to reconstruct defects of the hemiface, tongue, hypopharynx and esophagus [9–11]. As far as we know, there have been no reports on the use of pedicled TAAP flaps in tracheal reconstruction. This study aims to summarize the advantages and disadvantages as well as the significance of this flap in tracheal reconstruction of the neck by collecting clinical data regarding the use of pedicled TAAP flaps in tracheal reconstruction.

## Methods

### Patients

A retrospective review of 8 patients with cervical tracheal defects who underwent reconstructions with pedicled TAAP flaps between December 2017 and October 2019 at the Department of Otolaryngology, Head and Neck Surgery, West China Hospital of Sichuan University was performed. This retrospective review of medical records was approved by the Institutional Review Board of West China Hospital.

### Surgical process

All operations were performed under general anesthesia, and the anesthesiologist selected the appropriate intubation method based on the preoperative auxiliary examination. Subsequently, the surgeon completed the resection of the tracheal lesion and used pathologic examination to ensure tumor-free margins. In addition, cervical lymph node dissection was performed according to the disease type. After resection of the tracheal lesion, the defect was exposed and measured to prepare for flap harvest.

### Flap harvest and reconstruction

**Fig. 1:**
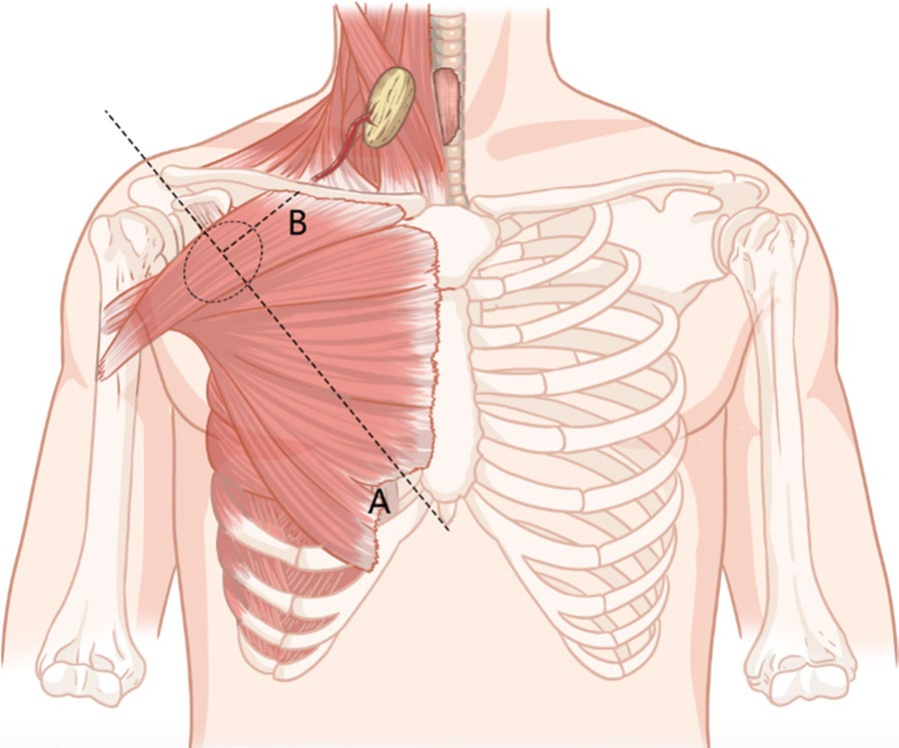
Design of pedicled thoracoacromial artery perforator flap for tracheal reconstruction

**Fig. 2:**
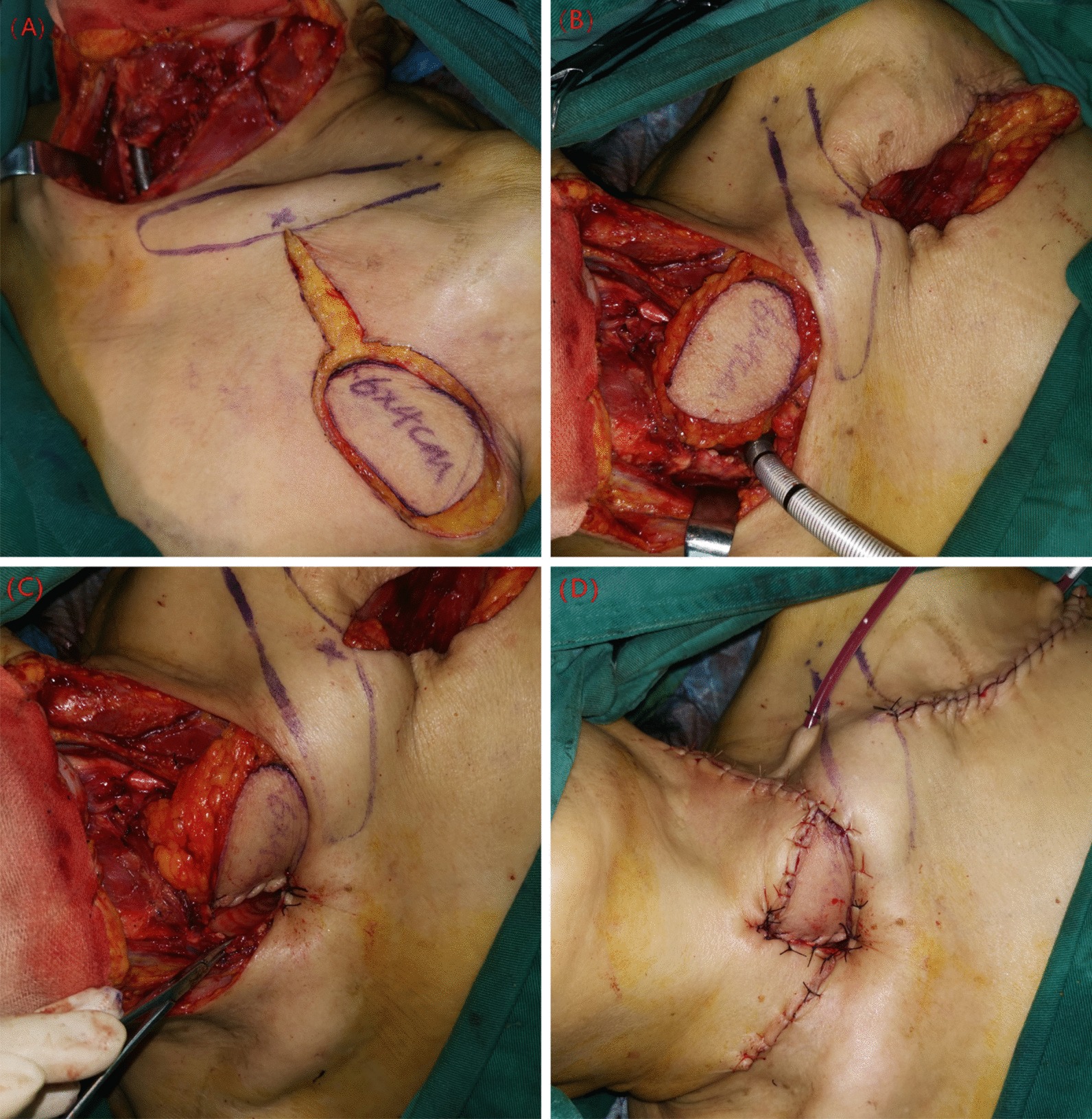
Reconstruction process. **a** The flap was designed according to the size of tracheal defect. **b** Flap has been elevated to defect through a tunnel under the clavicle. **c**, **d** The flap was sutured to the trachea and neck skin as for a permanent tracheostomy

The flap harvesting technique has been described previously [10] and is described as follows. Preoperatively, an intersection was made by a line joined from the acromion to the xiphoid process and a perpendicular line drawn from the midclavicular point. Doppler was used to probe the TAAP flap at this intersection (Fig. 1). Second, the flap was designed and obtained along the TAAP flap according to the size of the tracheal defect identified intraoperatively. Then, a tunnel under or over the clavicle was made to ensure that the flap could be elevated to the defect. It was necessary to avoid tension and distortion of the pedicle during elevation. After that, the flap was sutured to the neck skin and trachea for permanent tracheostomy. Finally, the donor site and surgical area of the neck were closed directly, and drainage tubes were placed separately (Fig. 2).

**Fig. 3:**
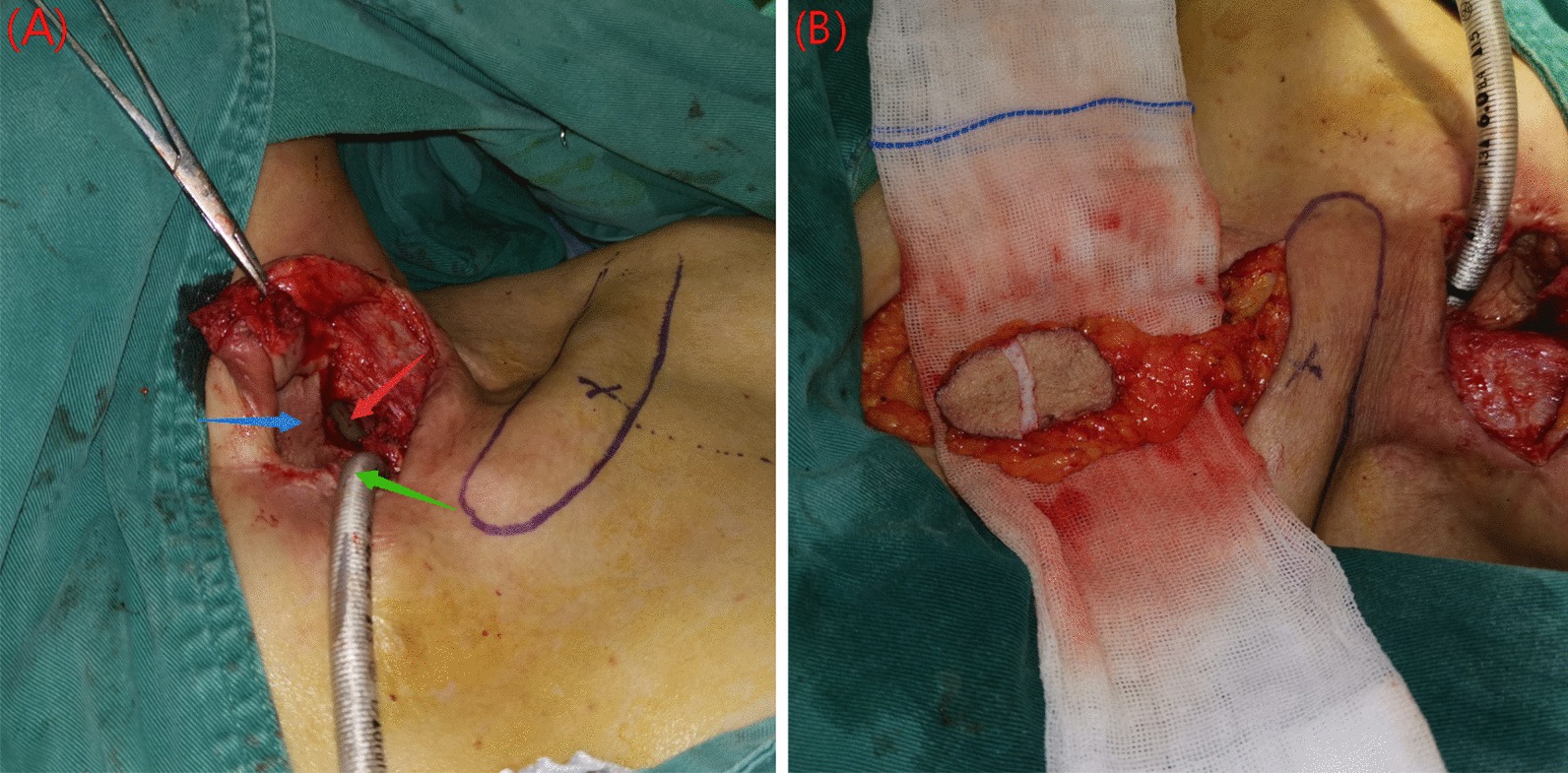
Double-island pedicled TAAP flap was used in tracheoesophageal fistula. **a** Tracheoesophageal fistula. The red arrow indicates the stomach tube, the green arrow indicates the tracheal catheter, the blue arrow indicates the flap of previous surgery. **b** Flap was sliced into two parts from the skin layer to be a double-island flap

In addition, a 72-year-old patient with tracheoesophageal fistula after thyroid cancer surgery received reconstruction with a double-island pedicled TAAP flap. The general harvest process of this flap was the same as mentioned above, but we added a step to divide the flap into two parts from the skin layer: one part was used to reconstruct the trachea, and the other part was used to reconstruct the esophagus (Fig. 3).

### Postoperative treatment and follow-up

Postoperatively, all patients received wound care, airway management, and anti-inflammatory drug administration. The status of the pedicled TAAP flap was inspected every hour through tracheostomal opening, and electronic laryngoscopy was performed to evaluate the flap thoroughly for 3 days. After discharge, regular follow-up was conducted to observe the flap condition and the occurrence of complications. Six months or more postoperatively, a second-stage tracheal reconstruction was conducted by direct suturing, natural healing or the placement of a local flap as we described previously [12]. Imaging examination, electronic laryngoscopy and the patient's complaint were usually used to evaluate the condition after the second-stage tracheal reconstruction.

## Results

### Demographics

There were 4 male patients and 4 female patients with a mean age of 62.4 years (range 22–83 years). Data regarding age, sex, BMI, comorbidities, past medical history, diagnosis and tracheal defects are summarized in Table 1.

**Table 1:** Demographics

Patient Number	Age	Sex	BMI	Comorbidities	Prior treatment	Diagnose	Defect length	Defect width	RLNI
1	83	Female	22.7	None	None	Thyroid carcinoma (T4N1M0)	7 TC	1/2 cir	Unilateral
2	22	Male	18.0	None	Thyroid surgery	Subglottic paragangliomas	6 TC and CC	1/2 cir	Unilateral
3	72	Female	20.5	Diabetes	Thyroid surgery and I131	Tracheoesophageal fistula	2 TC	1/4 cir	Bilateral
4	68	Male	31.9	Diabetes hypertension	None	Thyroid carcinoma (T4N0M0)	8 TC	3/4 cir	Unilateral
5	61	Female	23.7	Hypertension	None	Subglottic paragangliomas	7 TC and CC	1/2 cir	Unilateral
6	66	Male	21.3	COPD	Fibro bronchoscopic argon plasma coagulation	Tracheal squamous cell carcinomas (T4N1M0)	10 TC and CC	1/2 cir	No
7	73	Male	20.9	Hypertension asthma	Thyroid surgery	Thyroid carcinoma (T4N0M0)	6 TC and CC	3/4 cir	Unilateral
8	54	Female	20.8	None	Thyroid surgery and I131	Thyroid carcinoma (T4N0M0)	6 TC and CC	1/2 cir	Unilateral

*TC* tracheal rings, *COPD* chronic obstructive pulmonary disease, *CC* cricoid cartilage, *RLNI* recurrent laryngeal nerve involvement

### Surgical and clinical information

**Fig. 4:**
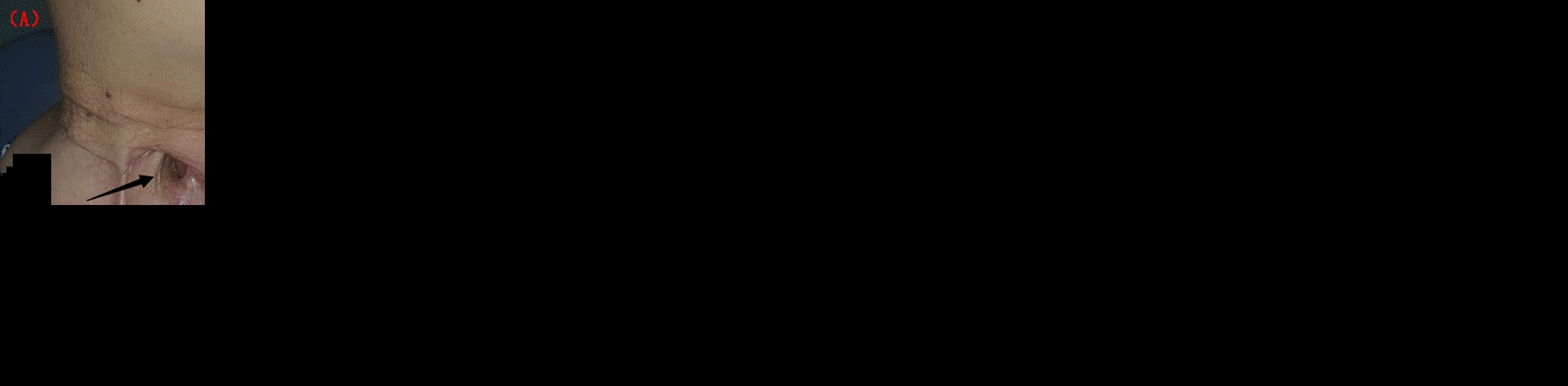
Postoperative follow-up. **a** The TAAP flap was in good condition (black arrow) under direct vision after the first stage tracheal reconstruction. **b** The donor site was well after operation. **c**, **d** CT showed the trachea after the second stage tracheal reconstruction with a local flap. The blue and red arrow indicates the pedicled TAAP flap, the green arrow indicates the local flap

All of the pedicled TAAP flaps in our study survived (Table 2, Fig. 4A).The flap size ranged from 2 cm × 5 cm to 4 cm × 10 cm (width × length, the length means being parallel to the axis of the arterial pedicle), and the size of each island of one double-island flap was 2 cm × 2.5 cm. The mean thickness was 0.6 cm, and the pedicle length varied between 6 and 9 cm (mean 7.9 cm). The mean time of flap harvest was 17 min (the time from the start of skin cutting to the flap being elevated to the defect). All patients returned to the ward instead of the intensive care unit after surgery. The average length of hospital stay after surgery was 7.5 days (range 6–11 days).

**Table 2:** Surgical and clinical information

Patient number	Flap size (width × length)	Flap pedicle length (cm)	Flap harvest time	Hospital stay time after surgery	Decannulation	Second-stage tracheal reconstruction	Follow-up
1	4 cm × 6 cm	9	17 min	9 days	Yes, 6 m	Direct suture	Well at 6 m
2	3 cm × 6 cm	8	16 min	8 days	Yes, 6 m	Natural healing	Well at 8 m
3	2 cm × 5 cm	6	15 min	6 days	No	NA	Dead at 4 m
4	5 cm × 7 cm	7	22 min	7 days	Yes, 7 m	Local flap reconstruction	Well at 13 m
5	4 cm × 7 cm	7	17 min	11 days (Low T3 syndrome)	Yes, 6 m	Local flap reconstruction	Well at 12 m
6	4 cm × 10 cm	8	16 min	6 days	Yes, 6 m	Direct suture	Well at 13 m
7	6 cm × 6 cm	9	16 min	7 days	No	NA	Well at 3 m
8	5 cm × 7 cm	9	17 min	6 days	Yes, 11 m	Local flap reconstruction	Well at 20 m

*NA* not available

### Follow-up and complications

The average follow-up was 9.9 months (range 3–20 months), 1 patient died due to distant metastasis of anaplastic thyroid carcinoma 4 months after surgery, 2 patients with thyroid cancer accepted I131 treatment after surgery, and 1 patient with tracheal squamous cell carcinoma received radiotherapy. 5 patients chose to undergo closure of the tracheotomy opening via placement of a local flap or direct suturing, and a young patient chose natural healing. These patients never complained of difficulty breathing after closure of the opening. Postoperative imaging and endoscopic examination also showed the patency of new tracheal (Fig. 4c, d).All patients had normal swallowing function after surgery, and the patient with bilateral recurrent laryngeal nerve involvement had poor speech function.

Low T3 syndrome occurred in one patient after surgery, and she recovered with the help of an endocrinologist. Other patients had an uneventful course without any obvious complications. The donor area was directly closed in all patients (Fig. 4b).Three patients complained of numbness in the donor area. No patients complained about the appearance of the donor area or abnormal chest or shoulder movements.

## Discussion

With the development of reconstruction technology, various tracheal defect reconstruction methods have emerged. The main goal of tracheal reconstruction is still to restore completeness and maintain good respiratory efficiency. End-to-end anastomosis is suitable for reconstruction of a simple defect within 4 cm; otherwise, fistula and stenosis are likely to occur [13]. For large or complex tracheal defects, clinicians need to use certain tissues for reconstruction, and the tissues also provide protection for peripheral vasculature and nerves. Depending on the type of tracheal defect, these tissues not only need to properly provide good biomechanical properties but also need a higher survival rate and resistance to infection [4]. Axial flaps have a reliable blood supply, a high survival rate and strong anti-infection ability, so they are favored by clinicians. Depending on whether axial flaps are completely separated from the donor area, they can be divided into pedicled and free flaps, and all of them have advantages and disadvantages.

Using a free flap to reconstruct the defect, the vascular pedicle of the flap is anastomosed with the vessels in the recipient area. Compared with that of parotid, temporal, and hypopharynx defects, the reconstruction of tracheal defects is usually completed in a narrow space, so there are higher requirements for the freedom of tissue placement. Therefore, when there are blood vessels in the recipient area, free flaps may be suitable for tracheal reconstruction, which is also related to the length of the pedicle. Free radial forearm flaps, anterolateral thigh (ALT) flaps and posterior tibial artery perforator flaps have been successfully used for the reconstruction of tracheal defects [12, 14, 15], but they also have some disadvantages. ALT flaps are also ideal for head and neck reconstruction, as they are easy to harvest and provide rich tissue volumes and types. However, these flaps are relatively thick, so the value for tracheal reconstruction is limited. Although relevant techniques can thin these flaps, they have high requirements for surgeons [16]. Free radial forearm flaps with thin tissue is suitable for tracheal reconstruction, and posterior tibial artery perforator flaps were a suitable for tracheal reconstruction in our previous studies because of the thin, tough and hidden donor site. However, harvesting the flaps described above is complicated, and skin grafts are always required in the donor area. On the other hand, the application of free flaps to reconstruct tracheal defects generally increases the operation time and requires skillful surgeons and microsurgical techniques. In our study, most patients are older than 60 years old and suffer some comorbidities. Therefore, long-term surgery is unfavorable for them. In addition, some patients underwent neck surgery or I131 treatment previously, which may be a threat to the recipient vessels for free flaps.

Pedicled flaps are also classic for repairing head and neck defects; owing to the vascular pedicle, the freedom of position in pedicled flaps is not as good as that in free flaps. However, pedicled flaps usually have a stable blood supply and simple harvest process, and surgeons do not require microsurgical techniques. Pectoralis major myocutaneous flaps have always been favored for reconstruction because of their simple harvesting process and stable blood supply. Nonetheless, for tracheal defects, this type of flap is too bloated, and its influence on the appearance and function of the donor area may be unacceptable [17].

Pedicled TAAP flaps are thinner than pectoralis major myocutaneous flaps, and the influence of the donor area is lower. Secondly, compared with the neck flap (such as platysma transverse myocutaneous flap), TAAP blood vessels is not susceptible to the influence of neck dissection. Furthermore, compared with the ALT flap and posterior tibial artery perforator flap, the pedicled TAAP flap provides a less hair inner lining in our study, which helps the patient feel comfortable. However, whether TAAP flaps have hair or not is also strongly related to race and gender. In addition, a pedicled TAAP flap can be made into a double-island flap while reconstructing tracheoesophageal fistulas. Last but not least, it takes a short time to harvest the flap, as the average time in our study was 17 min. This is especially suitable for elderly patients or other patients with poor tolerance to long-term surgery.

Of course, no flap is perfect, and the pedicled TAAP flap also has some disadvantages. First, its placement is limited by the vascular pedicle. Secondly, scars on the chest and possible nipple asymmetry affect the cosmetic effect. Moreover, the caliber of the pedicled TAAP flap is too small to be detected quickly and protected easily. Furthermore, numbness in the donor area can cause discomfort. Finally, we are still inexperienced in patients with rich adipose tissue in the chest, and the TAAP flap with rich adipose tissue may not be suitable for tracheal reconstruction owing to the difficult harvesting process and thickness.

There are some shortcomings in this study. The tracheal reconstruction by two-stage surgery seems to increase the complexity, but the closure of tracheal opening may be still needed after the one-time reconstruction, although this step is relatively simple. The difference of reconstruction strategy is limited in the assessment of the flap, but we will also use TAAP flaps for a one-time reconstruction of tracheal defects in the future. In addition, this study was completed in a retrospective manner, and the number of patients was relatively small. We intend to carry out a large number of prospective studies to better summarize the significance of pedicled TAAP flaps in tracheal reconstruction.

## Conclusion

Pedicled TAAP flaps may be a safe and reliable choice for the reconstruction of tracheal defects. These flaps have a thin thickness, a stable blood supply, a short harvesting time, and little influence on the donor area. Moreover, the flap can be designed as a double-island flap to reconstruct complex defects.

## Data Availability

All data generated or analysed during this study are included in this published article.

## Abbreviations

TAAP: Pedicled thoracoacromial artery perforator;
ALT: Anterolateral thigh
